# Cancer Cell Phenotype Plasticity as a Driver of Immune Escape in Melanoma

**DOI:** 10.3389/fimmu.2022.873116

**Published:** 2022-03-29

**Authors:** Valentin Benboubker, Félix Boivin, Stéphane Dalle, Julie Caramel

**Affiliations:** ^1^ Cancer Research Center of Lyon, Université de Lyon, Université Claude Bernard Lyon 1, INSERM, CNRS, Centre Léon Bérard, “Cancer cell Plasticity in Melanoma” team, Lyon, France; ^2^ Dermatology Unit, Hospices Civils de Lyon, CH Lyon Sud, Pierre Bénite Cedex, France

**Keywords:** cancer cell plasticity, EMT-like, melanoma, immune escape, immunotherapy resistance

## Abstract

Immunotherapies blocking negative immune checkpoints are now approved for the treatment of a growing number of cancers. However, even in metastatic melanoma, where sustained responses are observed, a significant number of patients still do not respond or display resistance. Increasing evidence indicates that non-genetic cancer cell-intrinsic alterations play a key role in resistance to therapies and immune evasion. Cancer cell plasticity, mainly associated with the epithelial-to-mesenchymal transition in carcinoma, relies on transcriptional, epigenetic or translational reprogramming. In melanoma, an EMT-like dedifferentiation process is characterized by the acquisition of invasive or neural crest stem cell-like features. Herein, we discuss recent findings on the specific roles of phenotypic reprogramming of melanoma cells in driving immune evasion and resistance to immunotherapies. The mechanisms by which dedifferentiated melanoma cells escape T cell lysis, mediate T cell exclusion or remodel the immune microenvironment will be detailed. The expanded knowledge on tumor cell plasticity in melanoma should contribute to the development of novel therapeutic combination strategies to further improve outcomes in this deadly metastatic cancer.

## Introduction

Immunotherapies blocking negative immune checkpoints were first approved for the treatment of metastatic melanoma and are now widely used for the treatment of a growing number of cancers, albeit with variable efficacy (1). Despite spectacular efficacy of immune checkpoint blockade (ICB) (anti-CTLA-4 and anti-PD-1 antibodies) in a subset of metastatic melanoma patients (2), primary (i.e. at the onset of treatment) or acquired resistance (i.e. after initial response) still occurs in 60% of patients (3).

The mechanisms of resistance to immunotherapies targeting inhibitory immune checkpoints, include both host extrinsic and intrinsic factors, the mechanisms of which are becoming increasingly clear (4). Predictive features of response by the tumor immune microenvironment have gained notoriety (5), particularly CD8^+^ T cell infiltration (6–8) and the presence of tertiary lymphoid structures (9–11), emerging as promising factors of response. Conversely, the presence of regulatory T cells (Tregs), myeloid-derived suppressor cells (MDSC), M2 macrophages, and other inhibitory immune checkpoints, contribute to inhibiting anti-tumor immune responses (3, 12–14). However, the emergence of additional tumor parameters has improved our predictive capacity. Indeed, tumor cell-intrinsic alterations have been associated with resistance to immunotherapy in melanoma. Namely, JAK1/2 mutations were associated with decreased Interferon-γ (IFN-γ) sensitivity of melanoma cells (15), and mutations in the gene encoding beta-2-microglobulin (B2M) to loss of surface expression of MHC-I and decreased antigen presentation (16). Oncogenic alterations of melanoma cells, including activation of the WNT-β-catenin (17, 18), MAPK (19), CDK4-CDK6 (20) pathways, or loss of PTEN expression (21–23) have also been associated with T cell exclusion and immune resistance in mouse models and human melanoma samples. Altogether, these tumor-intrinsic genetic alterations define the tumor mutational burden (TMB), the value of which to predict response to ICB was confirmed recently in a pan-cancer meta-analysis of over 1,000 patients, including 5 melanoma datasets (24).

However, growing evidence suggests the contribution of tumor cell-intrinsic non-genetic mechanisms, akin to the epithelial-to-mesenchymal transition (EMT), in the acquisition of resistance to immunotherapy in melanoma (25–29). EMT is an embryonic reversible cell plasticity process, by which an epithelial cell loses its polarity and adhesion to other cells, while gaining motility and mesenchymal features (30). EMT is required during the delamination of the embryonic neural crest, from which melanoblasts, the progenitors of melanocytes, originate (31, 32). This process is driven by intense transcriptional, epigenetic and translational reprogramming, involving EMT-associated transcription factors (EMT-TFs) (33, 34). Aberrant reactivation of EMT has been thoroughly characterized in carcinomas, as a multi-step dedifferentiation process driving metastasis, drug resistance and disease recurrence (35, 36). More recently, emerging evidence showed that this process is not limited to epithelial cancers, since EMT-like transitions have been described in non-epithelial malignancies, such as glioblastoma and neuroblastoma (37–40). The term of phenotype switching is therefore used to refer to EMT-resembling plasticity in non-epithelial cancers.

Aberrant activation of EMT factors is increasingly reported to contribute to immune evasion in various carcinoma models (41–44). In contrast, their contribution in the context of melanoma has been poorly studied, while they display cell-type specific roles in this neural crest-derived cancer (45, 46). In this review, we will focus on the impact of non-genetic mechanisms related to melanoma cell phenotype switching during immune escape and resistance to immunotherapies. After a brief overview of EMT-like transcriptional, epigenetic and translational mechanisms, we will review recent data highlighting how melanoma cell plasticity may impact anti-tumor immunity, including the loss of melanocytic antigens and other cell autonomous mechanisms that enable melanoma cells to evade cytotoxic CD8^+^ T cell lysis. In addition, we will discuss how melanoma cell dedifferentiation can reprogram the immune tumor microenvironment, mainly through the secretion of inflammatory cytokines and chemokines. Metabolic rewiring of melanoma cells, which also plays a major role in their interactions with the tumor microenvironment [already reviewed in Avagliano et al. (47)] will not be addressed. A better understanding of the mechanisms by which phenotypic adaptations of melanoma cells contribute to the acquisition of resistance to immunotherapies, will provide the basis for the development of new combination therapies.

## EMT-Like Phenotype Switching In Melanoma

Melanoma cells undergo reversible phenotype switching between a proliferative/differentiated phenotype and an invasive/stem-like state. Microphtalmia-associated transcription factor (MITF), the master regulator of melanocytic differentiation (48) was described as the major driver of phenotype switching. Indeed, decreased MITF expression promotes the transition towards a more invasive state, with stem-like features (49–51). Up-regulated expression of the AXL receptor tyrosine kinase, led to the definition of the MITF^low^/AXL^high^ phenotype (52). Recent analyses of tumor heterogeneity at the single cell level (20, 53, 54) further refined this phenotype switching model, with the description of intermediate states, including a neural crest stem cell-like (NCSC) phenotype [reviewed in (55, 56)]. The deciphering of the transcriptional and epigenetic mechanisms regulating these transitions is ongoing (57, 58). Indeed, analyses of the H3K27 acetylation pattern highlighted a major role for MITF and SOX10 transcription factors in the regulation of melanocytic enhancers, and AP-1 was described as the major regulator of mesenchymal-like state enhancers (59, 60).

Strikingly, EMT-TFs were shown to participate in the regulation of melanoma cell plasticity, with a specific expression pattern compared to that of carcinoma (61) [reviewed in (45)]. Our team demonstrated that ZEB2 and SNAIL2 are highly expressed in MITF^high^ proliferative melanoma cells, whereas decreased expression of ZEB2 and SNAIL2 and aberrant activation of ZEB1 and TWIST1 promoted phenotype switching towards an invasive, dedifferentiated and MITF^low^ phenotype (46). Notably, ZEB1 increases the expression of NCSC markers, such as NGFR (62), a major regulator of phenotype switching in melanoma (63).

Dynamic transcriptional regulation of EMT-TFs (ZEB1, TWIST1, SNAIL) and genes associated with invasive phenotypes (SOX9, POU3F2) is achieved through a bivalent promoter configuration, displaying both permissive H3K4me and repressive H3K27me histone marks; this “poised” state enables rapid induction of gene expression (64). In this context, the histone methyltransferase EZH2, the effector subunit of the PRC2 polycomb complex, was shown to promote EMT-like melanoma cell plasticity (65). An additional level of regulation of phenotype switching is provided at the translational level through the eIF4E/F translation initiation complex (66, 67). Dysregulated mRNA translation by aberrant activation of the MNK1/2-eIF4E axis plays a critical role in progression to an invasive state (68). Phosphorylation of eIF4E selectively increases the translation of a subset of mRNAs encoding proteins including NGFR or SNAIL (69). In addition, micro-environmental cues were shown to induce phenotype switching in melanoma cells *in vitro*, such as TNFα (25), TGF-β (70) and hypoxia (71), all known inducers of EMT in carcinoma models (30, 35).

Overall, our understanding of the EMT-like phenotype switching in melanoma has improved over the last decade, supporting its role in sustaining intra-tumoral heterogeneity [reviewed in (72, 73)]. Concomitantly, several studies gathered evidence for the role of melanoma cell-intrinsic plasticity in the resistance to BRAF^V600E^ targeted therapy (53, 57, 59, 62, 74), and reprogramming towards a NCSC phenotype was proposed as an adaptive response to targeted therapy [reviewed in (75)]. Emerging evidence sustaining the role of melanoma cell phenotype switching in immune resistance will be detailed below.

## Decrease in Tumor Cell Immunogenicity

### Loss of Melanoma Differentiation Antigens

Since the emergence of immunotherapy, considerable attention was given to the expression of melanoma differentiation antigens (MDAs) and their role in eliciting anti-tumor immunity. These tumor-associated antigens (TAAs), including but not limited to Melan-A/MART-1, gp100, TRP1/2 and tyrosinase, are mainly expressed by melanocytic lineage cells under the control of MITF (76). Expression of these melanoma antigens is known to vary greatly between individuals, and at inter- and intra-tumoral levels in the same patient (77), although this does not seem to be attributable to direct genetic alterations or defects in antigen processing (78). Several studies confirmed the immunogenicity of these TAAs, demonstrating that their targeting by the immune system induced tumor shrinkage in mice and melanoma patients (79–82). A more recent study confirmed the role of these TAAs in the response to PD-1 blockade therapy in melanoma (83). This work by Riaz and colleagues showed that MDA-expressing cell populations are depleted in biopsies of ICB-responding patients after treatment. Intra-tumor T cell receptor (TCR) sequencing revealed a clonal expansion of T cells, consistent with a tumor antigen-specific immune response in ICB-responding patients (83).

In their pioneering study, the team of T. Tüting demonstrated in murine models that melanoma tumor cells resisting immunotherapy, based on CD8^+^ T lymphocytes adoptive cell transfer (ACT), undergo a dedifferentiation process, coordinating the loss of melanoma differentiation markers and the gain of the neural crest marker NGFR (25). Interestingly, the treatment of human melanoma cells with TNFα alone recapitulated the phenotype switch observed in their *in vivo* model. A similar inflammation-induced dedifferentiation was recently reported in human samples, in a longitudinal study documenting the follow-up of a metastatic melanoma patient treated with MART-1-targeting cytotoxic T-lymphocyte ACT (28). The analysis of tumor biopsies collected before and on-treatment showed a decrease in MDA expression after treatment, with an enrichment of NGFR-expressing tumor cells in lesions collected during tumor progression. The authors suggested that in regressing lesions, the decrease in differentiation antigens may be due to their targeting by T cells, whereas the loss of MDA in progressing lesions would occur through dedifferentiation in response to immune pressure. Moreover, the authors demonstrated that conditioned medium from engineered T cells is sufficient to decrease the expression of MDA and stimulate NGFR expression in human melanoma cells. *In vitro*, melanoma cells resistant to MART-1-specific T cells lack the expression of MDA, and display an enrichment of the neural crest-like and undifferentiated melanoma transcriptomic signatures defined by Tsoi and colleagues (57, 84).

Our team previously reported that ZEB1 expression in melanoma cells induced the repression of MITF, and was associated with a significant decrease in differentiation antigens including MLANA, tyrosinase and MC1R, while simultaneously enhancing NGFR expression (46, 62), highlighting the mutually exclusive expression of EMT-TFs and MDA. Others found that EZH2 epigenetically silenced MDA expression under TNFα stimulation, while inducing ZEB1 (65). Preventing phosphorylation of eIF4E in *BRAF^V600^*; *PTEN* KO transgenic mouse models also results in the increased expression of MART-1 and gp100 by tumor cells and their recognition and eradication by CD8^+^ T cells (68). Phospho-eIF4E was also negatively correlated with the expression of MART-1 in patient samples.

**Figure 1:**
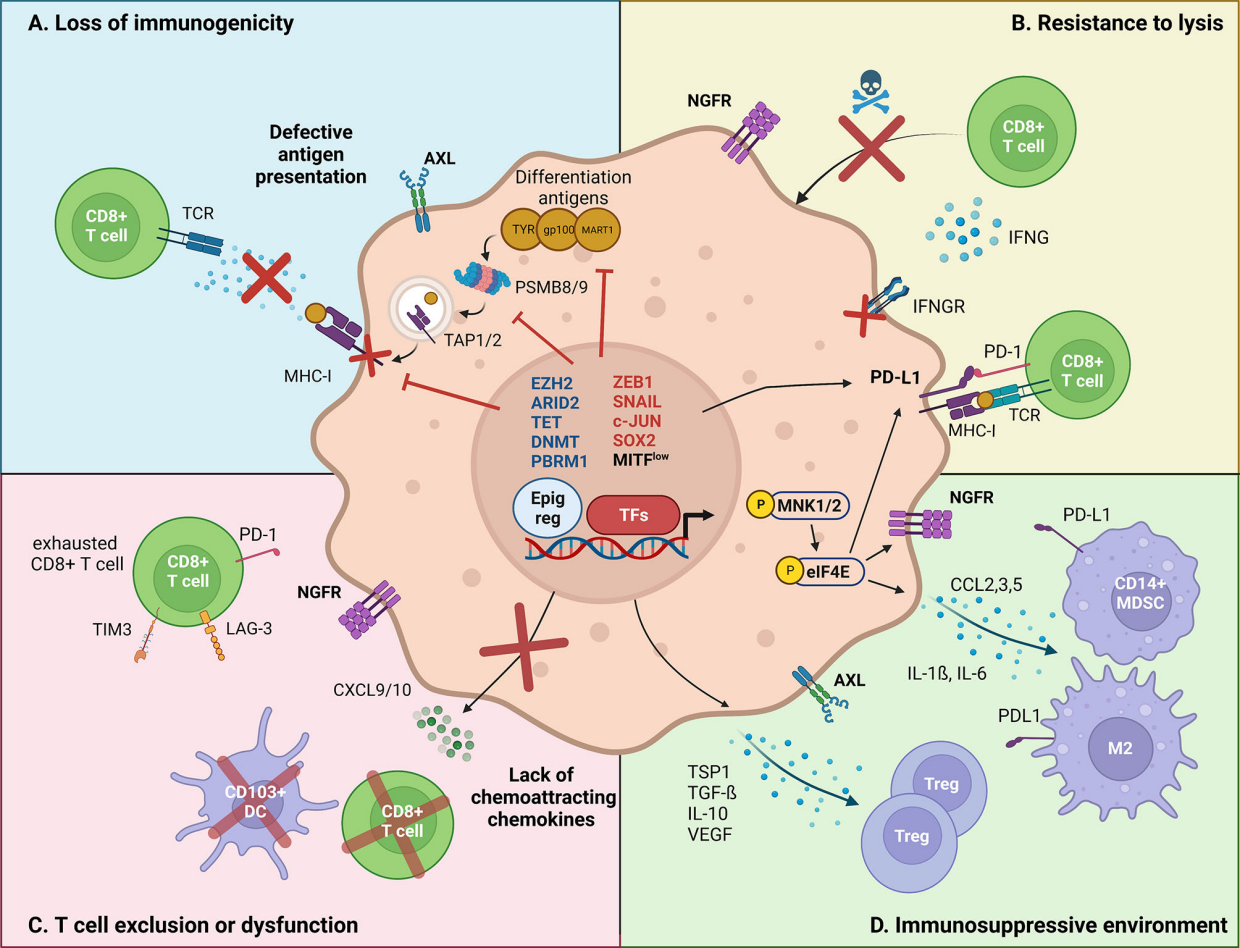
Major mechanisms by which melanoma cell-intrinsic pathways impact the crosstalk with the immune tumor-microenvironment. Melanoma cell-intrinsic pathways can mediate immune escape through transcriptional (TFs, transcription factors), epigenetic (Epig reg, epigenetic regulators) and translational mechanisms. **(A)** Loss of immunogenicity. Dedifferentiated melanoma cells express lower levels of melanoma differentiation antigens, such as TYR, gp100 and MART-1. Increased ZEB1 expression transcriptionally represses the expression of such markers. Immunogenicity is further decreased by transcriptional repression of the antigen presentation machinery (TAP1/2, PSMB8/9, MHC-I) by EZH2, and the transcriptional silencing of retroelements by SETDB1 and KDM5B. **(B)** Resistance to lysis. Increased PD-L1 expression allows dedifferentiated melanoma cells to inhibit the cytotoxic action of CD8^+^ T cells. Evidence suggests that PD-L1 expression is regulated both at transcriptional (DNA methylation) and at translational levels (CMTM6/eIF4E). Moreover, NGFR/BDNF and SOX2 render dedifferentiated melanoma cells inherently resistant to T cell-induced lysis, although the mechanisms remain unclear. Finally, epigenetic silencing of IFNγ-response genes, by aberrant DNA methylation (decreased TET2, upregulated DNMT3A) and chromatin remodeling (ARID2B, PBRM1), promotes the insensitivity to T cell-induced extracellular queues. **(C)** T cell exclusion/dysfunction. Phenotype switching impacts the recruitment of effector T cells, *via* reducing the production of chemoattracting chemokines. In particular, ZEB1 has been shown to transcriptionally impair the expression of CXCL10, preventing CD8^+^ T cell infiltration. Other epigenetic actors, such as ARID2 and PBRM1 were found to mediate a similar effect. Additionally, NGFR^high^ melanoma cells are associated with poor TILs infiltrate. **(D)** Immunosuppressive microenvironment. Dedifferentiated melanoma cells have been shown to induce an immunosuppressive and pro-tumoral immune microenvironment. Namely, the MNK1/2-eIF4E axis was shown to increase the translation of CCL2 and CCL5, which attract myeloid-derived suppressor cells (MDSC) and M2 macrophages. Additionally, the EMT-TF SNAIL stimulates the transcription of immunomodulating cytokines such as TSP1 and TGF-β, resulting in an increased infiltration of regulatory T cells.

Overall, melanoma cells often harbor TAAs, which can efficiently be targeted by cytotoxic T cells. The loss of these highly immunogenic differentiation antigens appears to be a non-genetic tumor cell-intrinsic immune escape mechanism consistently found in experimental settings (25, 84), as well as in patients (28), in response to immunotherapy (**Figure 1A**). However, the recognition of dedifferentiated melanoma cells by T cells specific for non­melanocytic antigens remained unaffected (25), indicating that these cells may still be killed through other cancer-associated antigens such as cancer testis antigens (CTA), including NY-ESO1, or neoantigens, originating from the degradation of cancer-specific mutated proteins (85).

### Loss of MHC Class I

Melanoma cell dedifferentiation has also been associated with alterations of antigen presentation, in particular in major histocompatibility (MHC) class I expression. MHC class I molecules are necessary for the recognition of TAAs by CD8^+^ T lymphocytes and can be induced by IFN-γ signaling. EMT has been associated with down-regulation of MHC class I in carcinoma models (41). A recent study conducted in the lab of A. Ribas found that the induction of the antigen presentation machinery in on-therapy melanoma biopsies was predictive of clinical response to ICB (86). Similar results were obtained in other cohorts, showing that lack of MHC class I expression is correlated with worse prognosis under ICB therapy (87). In this latter work, 43% of the cases harbored a complete loss of MHC class I expression, without any causal genetic event found. Evidence suggests a transcriptional regulation of the antigen presentation machinery, as genetic alterations in antigen presentation (B2M, HLA-A) and IFNγ signaling (IFNGR, STAT1 and JAK1/2) (16, 88) remain infrequent in melanomas and are not restricted to non-responding patients (89).

Combined RNA-sequencing and flow cytometry analyses of patient biopsies under anti-PD-1 treatment recently highlighted the negative correlation between MHC class I expression and melanoma cell dedifferentiation (89). In particular, transcriptomic analyses underlined a correlation between HLA-A (MHC class I) downregulation and SNAIL upregulation, a well-known EMT-TF. Patients categorized as HLA-A^low^ showed a dedifferentiated profile, with a MITF^low^/AXL^high^ phenotype, including downregulation of SOX10-regulated genes, TYR, and MLANA transcripts. The authors further demonstrated that MHC class I downregulation was recapitulated upon phenotype switching induced by TGF-β treatment *in vitro*. Interestingly, this coupling of dedifferentiation and MHC class I repression was observed in 31% of the immunotherapy-resistant patients in this cohort, corroborating the frequency observed by Rodig et al. (87).

The epigenetic regulator EZH2, was recently shown to coordinate melanoma cell dedifferentiation (associated with a gain in ZEB1 and NGFR) (65) with downregulation of MHC class I (HLA-A/B/C) and antigen presentation machinery (i.e. antigen processing genes TAP1/2 and immunoproteasome subunits PSMB8/9) (90). EZH2 is induced in response to immunotherapy (IL-2 or anti-CTLA-4) in tumors developed upon grafting of B16F10 or *Nras^Q61K^ Ink4a* ^-/-^ melanoma cells. Moreover, by using a chemical EZH2 inhibitor, the authors demonstrated that targeting EZH2 could synergize with immunotherapy in their murine models (90, 91). Other recent findings suggest that EZH2 could interact with DNA methyltransferases (DNMT) to mediate robust silencing of its target genes in melanoma, including the IFN-γ pathway (92). In melanoma patient biopsies, a methylome study revealed that DNA methylation on PRC2 target gene promoters is associated with poor MHC class I expression and lack of immune infiltration (93).

Moreover, a recent *in vivo* CRIPR-Cas9 screening in B16F10 cells highlighted the role of the histone methyltransferase SETDB1 in reducing the immunogenicity of murine melanoma cells, thus enhancing resistance to anti-PD-1 therapy (94). An independent study showed that SETDB1 cooperates with histone demethylase KDM5B/JARID1B to silence retroelements, which decreases antigen presentation in melanoma cells, efficiently promoting immune evasion (95). Interestingly, high KDM5B/JARID1B expression was previously reported in slow cycling therapy-resistant melanoma cells (96), and it is known that ZEB1 induces the upregulation of JARID1B in melanoma (62).

Altogether, although the precise role of EMT-inducing transcription factors in mediating down-regulation of MHC class I expression in melanoma cells is still lacking, tumor cell dedifferentiation and silencing of antigen presentation clearly appear to be intertwined. The EMT-like phenotype switching occurring in melanoma would therefore contribute to reducing the immunogenicity of tumor cells by concomitantly repressing melanoma differentiation antigens and the antigen presentation machinery, resulting in immune escape and resistance to ICB by avoiding T cell recognition (**Figure 1A**).

## Resistance to Lysis

### Upregulated PD-L1 Expression

Immune checkpoint ligands are key actors in the regulation of anti-tumor immune response. In particular, the expression of programmed cell death-ligand 1 (PD-L1/CD274) by tumor cells is a potent mechanism of immune escape by suppressing the activity of effector T cells expressing its receptor PD-1 (97) (**Figure 1B**). In carcinomas, a growing body of evidence suggests that EMT induces an upregulation of PD-L1 on tumor cell surface (98–100). Notably, the EMT-TF ZEB1 was shown to stimulate PD-L1 expression by repressing miR-200 in lung and breast cancer, leading to immune escape and metastasis (101, 102). Consistently, spontaneous lung tumors in mice with inactivated *Zeb1* harbor a significantly reduced PD-L1 expression at the invasive front, associated with a stronger anti-tumor immunity in this model (103).

PD-L1 expression displays a high inter- and intra-patient variability in melanoma, and the evaluation of PD-L1 alone as a predictive marker for response to anti-PD-1 remains debated (104). While PD-L1 is known to be induced by IFN-γ signaling in response to T cell activation and infiltration (105, 106), constitutive PD-L1 expression on tumor cells is observed in approximately 10% of melanomas (104, 107, 108). Interestingly, the characterization of melanoma cell lines with constitutive PD-L1 expression revealed a strong enrichment in EMT transcriptomic signatures compared to cell lines with inducible PD-L1 expression (108). Melanoma patients with constitutive PD-L1 expression (TIL^-^/PD-L1^+^) had a significantly worse median survival rate than patients with inducible PD-L1 expression (TIL^+^/PD-L1^+^) (107).

RNA-sequencing in PD-L1 null melanoma biopsies have uncovered a down-regulation of IFN-γ-induced genes compared to PD-L1-positive tumors (107). DNA methylation has recently been shown to mediate the repression of IFN-γ response pathways. In particular, PD-L1 expression is strongly negatively correlated to methylation levels of its promoter, both in cell lines (108) and in patient samples (109). Interestingly, the DNA methyltransferase DNMT3A was also shown to be negatively correlated to PD-L1 (108), and to promote dedifferentiation of melanoma cells (110). Furthermore, the repression of DNA demethylase TET2, which is also associated with phenotype switching (110, 111), participates in the resistance to anti-PD-1 therapy in B16 mouse models by silencing IFN-γ response genes such as PD-L1, CXCL9 and CXCL10 (112). Together, these results highlight the epigenetic silencing of the IFN-γ pathway in tumor cells as an immune escape mechanism.

While PD-L1 may be regulated at the transcriptional level, recent data highlighted a translational control through the translation complex eIF4E in melanoma (113) (**Figure 1B**). Genome-wide CRISPR-Cas9 screens additionally identified CMTM6 as a crucial mediator of PD-L1 translational regulation in a broad range of cancer cells including melanoma cells (114). CMTM6, which is expressed at the cell surface, increases PD-L1 protein pool without affecting its transcription levels. Indeed, CMTM6 associates with the PD-L1 protein, reducing its ubiquitination and increasing its half-life. EMT has also been shown to regulate surface PD-L1 *via* CMTM6 induction in breast cancer (115), though this link remains to be addressed in melanoma. eIF4E complex formation is associated with CD8^+^ T cell infiltration and inducible PD-L1 expression in patient samples correlated with response to immunotherapy in a cohort of 59 patients with metastatic melanoma treated with the anti-PD-1 monoclonal antibody pembrolizumab. While PD-L1 alone was not predictive, its inducible expression by CD8^+^ T cells through PD-1 PD-L1 proximity, predicted a better response (113).

### Escape From T Cell/NK Cell Cytotoxicity

The intrinsic properties of dedifferentiated melanoma cells favor resistance to T cell cytotoxicity, hence contributing to immune escape and resistance to ICB (84, 116). Indeed, MART-1 T cell-resistant melanoma cells were reported to harbor an NGFR^high^ dedifferentiated phenotype (84). NGFR, and its ligand BDNF, actively mediate this resistance to T cell-induced lysis, as their knock-down sensitized melanoma cells to MART-1 T cells. This study was the first to suggest a pro-survival role of NGFR rather than being only a dedifferentiation marker (84).

A CRISPR/Cas9 screening using co-culture experiments with melanoma cells and gp100-specific T cells identified the chromatin remodeling complex pBAF as an inducer of resistance to T cell killing by regulating chromatin accessibility on IFN-γ response targets (117). Knocking-out *Arid2* or *Pbrm1* also significantly increased PD-L1 expression (117).

Recently described stemness-associated mechanisms could also participate in the acquired resistance to T cell cytotoxicity by melanoma cells. SOX2, a stem-associated transcription factor, was recently reported to sustain the expression of late IFN-γ response immunosuppressive genes (PD-L1, IDO1), promoting resistance to T cell lysis in co-cultures (116). SOX2 silencing by the HDAC inhibitor SAHA synergized with anti-PD-1 therapy in mouse models. Interestingly, the authors found that SOX2 expression could segregate PD-L1^high^ patients into responding versus non-responding patients in published cohorts (116).

Although most studies focus on T cell-mediated lysis, melanoma cells may also develop mechanisms to escape from innate cytotoxicity triggered by natural killer (NK) cells. At the molecular level, melanoma cells may increase NK-protective MHC-I expression or induce a decrease in tumor-recognizing activating receptors on NK cells (118). Recent studies suggested that Integrin beta-like protein 1 (ITGBL1), a secreted protein, upregulated in MITF^low^ melanoma cells, inhibits NK cells cytotoxicity (119). However, the role of MITF might be more complex (120), since another study showed that MITF-expressing cells would also escape NK cell lysis, by regulating the expression of ADAM10, which cleaves the MICA/B family of ligands for NK cells (121). This is reminiscent of the previously described association of EMT with increased susceptibility to NK cell cytotoxicity in carcinoma models (122). Decreased MHC-I expression at the surface of mesenchymal cells, may indeed promote activation of NK cells by decreasing inhibitory signal. NK cells could also drive phenotype switching of melanoma cells (118). Overall, while the mechanisms of escaping from T cell-mediated lysis are increasingly characterized, the complex relationship between melanoma cells and NK cells will need further investigation.

## T Cell Exclusion or Dysfunction

In addition to escaping recognition and lysis by immune cells, tumor cells may also modify the composition and functionality of the immune microenvironment. CD8^+^ T cell recruitment and activation following treatment is a major parameter associated with the response to immunotherapy (83, 123) (**Figure 1C**). TGF-β, a well-known inducer of EMT (124), has been associated with the exclusion of cytotoxic T cells in carcinoma models (125, 126). In accordance, combined treatment with antibodies targeting PD-L1 and TGF-β was shown to induce CD8^+^ T cell infiltration and tumor regression. However, few studies are available in melanoma.

Alterations in various oncogenic pathways have also been associated with T cell exclusion or dysfunction in melanoma (21). Indeed, activation of the β-catenin pathway and loss of PTEN are two major independent and non-redundant mechanisms mediating T cell exclusion in metastatic melanomas (18, 22). A longitudinal follow-up of a patient with metastatic melanoma who relapsed upon interleukin-2 treatment revealed an activation of the β-catenin pathway, associated with the absence of infiltrating CD8^+^ T cells and decreased chemokine expression in the recurrent tumor (23). These findings provide evidence that tumor cell-intrinsic β-catenin activation induces an immune-deprived environment, that impairs immune control even in the setting of IL-2 immune stimulation. More recently, another study showed that serine/threonine­protein kinase PAK4, a WNT signaling mediator, was enriched in immunologically cold tumors from patients with melanoma resistant to anti-PD-1 immune checkpoint blockade. In multiple mouse models, genetic deletion or pharmacological inhibition of PAK4 resulted in reversal of resistance to anti-PD-1 therapy (17).

In addition, high NGFR expression in melanoma cells has been linked to immune exclusion in human melanoma samples (84). Indeed, NGFR expression by melanoma cells, as assessed by immunohistochemistry, was inversely correlated with the presence of CD8^+^ T cells. This was also evidenced at the intra-tumoral level in melanoma heterogeneous cases. These findings suggest that NGFR^high^ melanoma cells are not only resistant to T cell targeting but are also associated with poor T cell infiltration.

More recently, we investigated the role of the EMT-inducing transcription factor ZEB1 in immune evasion, and demonstrated its role in preventing T cell infiltration in melanoma (127). Multi-immunofluorescence spatial analyses of the immune infiltrates in human melanoma samples highlighted that high ZEB1 expression in tumor cells was associated with decreased CD8^+^ T lymphocyte infiltration. Gain- or loss-of-function experiments in BRAF or NRAS-mutated melanoma mouse models demonstrated that ZEB1 prevents the recruitment and the activation of CD8^+^ T cells. Mechanistically, ZEB1^high^ melanoma cells showed a defective secretion of T cell-attracting chemokines, including CXCL10, suggesting the intrinsic role of ZEB1 in regulating the secretome and subsequent immune cell attraction. ZEB1 directly binds to the promoters of T cell-attracting chemokines, including CXCL10 to repress their transcription. ZEB1-mediated T cell exclusion promotes immune evasion, as we showed that ZEB1 overexpression promotes resistance, whereas *Zeb1* knock-out improves the efficacy of anti-PD-1 immunotherapy in melanoma mouse models. Overall, our data indicate that CXCL10 partially accounts for ZEB1-mediated CD8^+^ T cell deficiency, and suggest additional mechanisms may contribute to immune escape. Moreover, ZEB1 may regulate CD8^+^ T cell-dependent tumor growth at least in part independently of MITF-mediated phenotype switching, since similar phenotypes were observed in MITF^high^ and MITF^low^ backgrounds.

Other melanoma-intrinsic pathways have also been associated with T cell deficiency. Knocking-out the epigenetic regulators *Arid2* or *Pbrm1* in B16F10 melanoma cells significantly increased CXCL9/CXCL10 production and CD8^+^ infiltration *in vivo*, resulting in decreased tumor growth (117). SOX2 overexpression in B16F10 melanoma cells, in addition to mediating resistance to T cell killing, decreased infiltration of CD8^+^ T cells, albeit the underlying mechanisms remain unclear (116). Although not related to a phenotype switch in the immune-competent melanoma mouse models used, *Sox10* knock-out was also shown to reduce melanoma tumor growth in a T cell-dependent manner (128).

The link between the melanoma differentiation state and T cell exclusion/dysfunction was recently nicely addressed in novel mouse models, that will be useful tools to further dissect melanoma cell-intrinsic mechanisms of immune escape. Four immunocompetent melanoma mouse models differing in their differentiation status were generated and extensively characterized in relation with their differential sensitivity to immunotherapy (129). These models, based on different genetic backgrounds, showed similarities with previously described differentiation signatures (57); the M1 (NCSC-like) and M2 (undifferentiated) models were resistant to anti-CTLA-4 and anti-PD-1, while more differentiated models, M3 (melanocytic) and M4 (transitory), displayed partial response to ICB. The capacity to present antigens through MHC-I and to activate CD8^+^ T cells was maintained in all models. Importantly, resistant M2 tumors which displayed an undifferentiated signature, harbored only few T cells with a high exclusion score, except for Treg (130). However, TIL abundance in untreated tumors was not sufficient to explain ICB response, since M1 tumors were resistant despite a high CD8^+^ T cell/Treg ratio. CD8^+^ T cells in M1 tumors were shown to display a high dysfunctional score as determined by TIDE (T cell dysfunction and exclusion score), with increased expression of PD-1, LAG-3 and TIM3 exhaustion markers. Moreover, these studies highlighted the importance of assessing the myeloid cell compartment in addition to T cells: pro-tumor macrophages (especially PD-L1^+^) were enriched in M2 and M1 tumors, while dendritic cells (DC) and NK cells were further reduced in M2 tumors. Overall, melanoma cell dedifferentiation is associated with decreased T cell abundance and function, but may also impact the recruitment or function of other immune cell populations.

## Induction of anImmunosuppressive Microenvironment

Previous studies in carcinoma have shown that expression of EMT-TFs (notably ZEB1, SNAIL and TWIST1) is associated with the recruitment of immunosuppressive cells, including regulatory T cells, myeloid-derived suppressor cells and tumor-associated macrophages (TAMs) (41–43, 131–133).

Dedifferentiation of melanoma cells has been associated with an immunosuppressive microenvironment (**Figure 1D**). Namely, the dedifferentiated MITF^low^/c-Jun^high^ melanoma phenotype in human melanoma samples, was associated with increased infiltration by myeloid immune cells, as assessed by CD14 immunohistochemistry (134). Increased Gr-1^+^ myeloid cell recruitment was confirmed in dedifferentiated Hgf-Cdk4^R24C^ mouse melanoma models obtained after escape from ACT against the melanocytic antigen gp100. In an ACT model directed against the melanosomal protein RAB38, recurrent melanomas with dedifferentiated features also showed increased myeloid cell recruitment, related to increased secretion of myeloid cell-attracting chemokines (CCL2, 3, 5) (135). Overall, several studies reported that the MITF^low^ phenotype is associated with elevated NF-kB activity, and increased expression/secretion of pro-inflammatory cytokines IL-1β, IL-6 and CCL2 chemokine that may contribute to myeloid cell recruitment (136).

SNAIL was shown to favor an immunosuppressive microenvironment in melanoma models (137). SNAIL overexpression in melanoma cells *in vivo* promotes the recruitment of Tregs and impairs dendritic cell maturation in a process involving the immunosuppressive cytokines thrombospondin (TSP1) and TGF-β. Furthermore, blocking SNAIL with siRNA or anti-TSP1 monoclonal antibodies inhibited tumor invasion by relieving this immunosuppressive mechanism. In addition to promoting CD8^+^ T cell deficiency, ZEB1 overexpression in melanoma mouse models was also associated with an increased frequency of Treg, although at a later stage. No significant modifications in the overall frequency of macrophages and DCs were observed in these models upon ZEB1 expression, although more precise phenotyping of these immune populations will be required (127).

Moreover, CD133^+^ melanoma cancer stem cells (CSC) in the B16F10 model were associated with increased abundance of immunosuppressive cells, including Tregs, MDSCs, and M2 macrophages (138). Interestingly CD133^+^ CSC harbored an increased TGF-β signature that was regulated by miR-92 through an integrin-dependent TGF-β activation.

Recently, the MNK1/2-eIF4E axis was also involved in the regulation of melanoma plasticity and anti-tumor immune response by promoting a suppressive tumor microenvironment (68). In a *BRAF^V600^*; *PTEN* KO mouse melanoma model in which eIF4E cannot be phosphorylated (eIF4EKI), they showed an increase in the secretion of many cytokines linked with the expansion, recruitment (such as CCL2, CCL12, and CCL5), and function (such as MMP-9) of immunosuppressive cells such as MDSCs. Immune phenotyping showed a significant infiltration of cytotoxic CD8^+^ T cells linked to a decrease in monocytic MDSCs in eIF4EKI compared with eIF4EWT melanomas (68). Combined treatment with MNK1/2 inhibitors and anti-PD-1/PD-L1 demonstrated its efficacy in several mouse melanoma models, arguing in favor of defining a new strategy to inhibit melanoma plasticity and improve response to anti-PD-1 immunotherapy.

Altogether cancer cells use several ways to promote immune escape notably by inducing an immunosuppressive microenvironment through phenotype switching. Strategies aiming at targeting key players of melanoma cell plasticity could thus improve response to immune checkpoint therapy (**Table 1**). Such combination strategies may not only be addressed in preclinical mouse models, but their efficacy may be further evaluated *ex vivo* on fresh human melanoma slices (142), as nicely reported recently. This will also allow the validation of predictive biomarkers.

**Table 1:** Different strategies targeting melanoma cell plasticity-associated players to potentiate ICB efficacy.

Target	Strategy	Combination	Status	Clinical trial	Phase	References
**ACT**	MART-1 T-cells	Aldesleukin	C	Metastatic melanoma	II	NCT00910650 (139),
	TGFß-resistant, NGFR transduced T-cells	Aldesleukin	R	Stage III or Metastatic melanoma	I	NCT01955460
	CXCR2 and NGFR transduced T-cells	Aldesleukin	NR	Stage III or Metastatic melanoma	I/II	NCT01740557
**TGFβ**	SRK-181	Anti-PD1	R	Solid tumors	I	NCT04291079
			POC	/	/	(140)
**AXL**	AXL-107-MMAE	BRAF + MEK inhibitors	POC	/	/	(141)
	BGB324	Pembrolizumab or Dabrafenib + Trametinib	R	Advanced non-resectable (Stage IIIc) or Metastatic (Stage IV) Melanoma	Ib/II	NCT02872259
	INCB081776	Nivolumab	R	Solid tumors	Ia/Ib	NCT03522142
**EZH2**	shRNA, GSK503	Anti-CTLA4	POC	/		(90)
	Tazemetostat	BRAF + MEK inhibitors	R	Metastatic melanoma	I/II	NCT04557956
**MNK1/2**	Tomivosertib eFT508	/	C	Solid tumors	I/II	NCT02605083
		Anti-PD1	U	Solid tumors	II	NCT03616834
	SEL201	Anti-PD1	POC	/	/	(68)
**HDACs**	SAHA	Anti-PD1	POC	/	/	(116)
	Panobinostat	Ipilimumab (anti-CLTA4)	NR	Unresectable stage III/IV Melanoma	I	NCT02032810
	Entinostat	Pembrolizumab (anti-PD1)	R	Non-inflamed stage III/IV melanoma	II	NCT03765229
	Tinostamustine	Nivolumab (anti-PD1)	R	Advanced melanoma	Ib	NCT03903458
**SETDB1**	Knock-out	Anti-PD1	POC	/	/	(94)
**ARID2, PBRM1**	Knock-out	Anti-PD1	POC	/	/	(117)
**TET2**	Vitamin C	Anti-PD1	POC	/	/	(112)
**ZEB1**	Knock-out	Anti-PD1	POC	/	/	(127)

Both clinical trials and proof of concept experiments in mouse models are indicated.

ACT, Adoptive Cell Transfer; R, Recruiting; NR, Not recruiting; U, Unknown; C, Complete; POC, Proof of concept.

## Melanoma Cell-Intrinsic Predictive Markers of Resistance to Immune Checkpoint Blockade

Previous reports have suggested that EMT or mesenchymal signatures were associated with a lack of response to ICB in carcinoma (36, 42, 43). As described in this review, preclinical studies in mouse models highlighted the contribution of many non-genetic mechanisms in the resistance to ICB in melanoma, but only few studies demonstrated the predictive value of these parameters in large human cohorts of ICB-treated patients. Difficulties reside in the inability to identify robust signatures/biomarkers of response to ICB from bulk RNA-Seq data of tumors at baseline, before treatment.

By comparing responding versus non-responding pre-treatment tumors Hugo et al. first described an innate PD-1 resistance (IPRES) gene signature, including upregulation of mesenchymal markers (such as AXL, WNT5A, or TWIST2) and immunosuppressive cytokines (27). Another ICB pre-treated melanoma cohort showed that TGFB1^low^ and SOX10^high^ expression was associated high cytotoxic T lymphocyte levels and a better overall survival (130). RNA-Seq data of patients treated with ICB revealed that the tumor-intrinsic NGFR signature predicts resistance to anti-PD-1 therapy before treatment and is further increased upon treatment (84). However, transcriptomic signatures such as IPRES have not been able to consistently predict response to anti-PD-1 in several independent melanoma cohorts (83, 89, 123, 143). One limitation resides in the fact that part of these melanoma samples received BRAF inhibitors (BRAFi) prior to ICB. Indeed, previous treatment with BRAFi may be a confounding parameter, as BRAFi is known to induce ZEB1 expression and promote a higher mesenchymal score (62). In line with these observations, a recent study demonstrated in melanoma patients and mouse models, that tumors relapsing after targeted therapy with MAPK pathway inhibitors are cross-resistant to immunotherapies (144). Resistance to targeted therapy in melanoma leads to activation of a cancer cell-intrinsic signaling program with enhanced and altered transcriptional output associated with an immunosuppressive tumor microenvironment, characterized by a lack of functional CD103^+^ DCs and T cells. Inhibition of the MAPK pathway in BRAFi-resistant tumors restored tumor infiltration and maturation of CD103^+^ DCs, reduced suppressive myeloid cells, increased T cell infiltration, and re-sensitize cross-resistant tumors to immunotherapy.

More recently, Pérez-Guijarro et al. defined a 45-gene melanocytic plasticity signature (MPS), consisting of 33 upregulated and 12 downregulated genes, in undifferentiated/NCSC resistant melanoma mouse models and validated its efficacy in predicting response in human cohorts. While the precise molecular mechanisms involved remain to be functionally addressed, they demonstrated that combination of MPS and TIDE (T cell dysfunction and exhaustion) signatures was a stronger predictor (129). Overall, this highlights the interest of combining cancer cell-intrinsic and immune parameters in order to better predict patient survival in response to ICB.

However, there may still exist limitations/biases of using bulk RNA-Seq approaches, since EMT-associated genes are not only expressed by cancer cells but also by cells from the tumor micro-environment, including cancer-associated fibroblasts (CAFs), endothelial cells, and immune cells. This constitutes an important confounding parameter in bulk RNA-Seq analyses, especially in samples from lymph nodes metastases. This problem can be overcome through single-cell RNA-seq analyses (20, 54) or through spatial analyses of tumors (127). By using scRNA-seq data, Tyler and Tirosh recently defined cancer or stromal cell-specific mesenchymal signatures. Such a deconvolution tool would allow to circumvent the confounding effect of stromal cells in bulk RNA-Seq data (145). In parallel, we already demonstrated the power of multiplexed immunofluorescence analyses to specifically analyze ZEB1 expression in melanoma cells, by excluding stromal and immune cells. Whether ZEB1/EMT-TF specific expression by melanoma cells may predict response to ICB in patient cohorts remains to be addressed.

Furthermore, since plasticity is a dynamic event, longitudinal follow-up of patients during immunotherapy, and not just baseline analyses, is required to comprehend the dynamic evolution of cancer cells and their microenvironment. In a recent study conducted in the group of G. Boland, a patient was followed for 9 years, collecting tumors from primary tumor, metastatic recurrence, pre-treatment, on-treatment, post-progression and at autopsy. This allowed them to establish an evolutionary dynamic map of resistance to ICB. Deconvolution of bulk RNA-Seq and highly multiplexed immunofluorescence characterized a dedifferentiated neural-crest (NGFR^high^) tumor population during resistance to immunotherapy. Moreover, they described distinct NGFR^high^ tumor cell distribution patterns, highlighting site-specific heterogeneity in tumor-immune interactions that will require further investigation (29).

## Conclusion and Future Perspectives

Throughout this review, we described several complementary mechanisms by which melanoma cell-intrinsic pathways can impact the crosstalk with the immune tumor microenvironment. Transcriptomic analyses in patient samples contributed to pinpointing that melanoma dedifferentiation markers may prove useful in combination with immune parameters to design a composite score for better predicting patients that may or may not respond to immunotherapy. In the future, the development of single cell spatial transcriptomic analyses, performed on samples biopsied in a longitudinal way upon treatment, will be crucial to precisely decipher the crosstalk between cancer cells and immune cells. A better understanding of these complex, intertwined interactions, will pave the way to novel combinatory treatments, to rescue resistance to ICB and improve metastatic melanoma patient care.

## Author Contributions

VB and FB contributed equally to writing and figure preparation. SD provided grant support and critical reading. JC wrote and supervised the whole process. All authors contributed to the article and approved the submitted version.

## Funding

The team is supported by the Lyon Integrated Research Institute in Cancer (SIRIC LYriCAN INCa-DGOS-Inserm_12563), the Institut Convergence PLAsCAN (ANR-17-CONV-0002), the ERiCAN program of Fondation MSD-Avenir (Reference DS-2018-0015), the Institut National contre le Cancer (INCA-DGOS PRTK Melpredict), ARC (Sign’it grant Birdman), the Ligue Nationale contre le Cancer, the Société Française de Dermatologie (SFD), the association Melarnaud and Vaincre le Mélanome. FB is supported by ITMO Cancer of Aviesan within the framework of the 2021-2030 Cancer Control Strategy, on funds administered by Inserm.

## Conflict of Interest

The authors declare that the research was conducted in the absence of any commercial or financial relationships that could be construed as a potential conflict of interest.

## Publisher’s Note

All claims expressed in this article are solely those of the authors and do not necessarily represent those of their affiliated organizations, or those of the publisher, the editors and the reviewers. Any product that may be evaluated in this article, or claim that may be made by its manufacturer, is not guaranteed or endorsed by the publisher.
